# Management of Hypertension With Non-pharmacological Interventions: A Narrative Review

**DOI:** 10.7759/cureus.43022

**Published:** 2023-08-06

**Authors:** Pratyusha Kodela, Monalisa Okeke, Sandeep Guntuku, Shanmukh Sai Pavan Lingamsetty, Eduard Slonovschi

**Affiliations:** 1 Internal Medicine, Shri B.M. Patil Medical College, Hospital and Research Center, Vijayapura, IND; 2 Internal Medicine, Drexel University College of Medicine, Philadelphia, USA; 3 Internal Medicine, Mamata Medical College, Khammam, IND; 4 Surgery, Universitatea de Medicină și Farmacie Iuliu Haţieganu Facultatea de Medicină, Cluj-Napoca, ROU

**Keywords:** alcohol, exercise, weight loss, intermittent fasting, dash diet, hypertensive emergency, coronary artery disease, elevated blood pressure, non pharmacological interventions, hypertension

## Abstract

Hypertension (HTN) is a condition that affects nearly half of the adult population in the United States. HTN is the elevation of blood pressure (BP) 130/80 mm Hg or higher. Untreated high blood pressure may increase the risk of myocardial infarction, stroke, and other serious complications. BP over 180/120 mm Hg with end-organ damage is called a hypertensive emergency. Despite advancements in medicine and treatment options, the global burden of HTN has been increasing due to the advancing age of the population and an increasing prevalence of obesity. Non-pharmacological management of HTN has gained prominence worldwide due to its additional benefits and positive impact on the overall health of individuals, having almost no side effects and reducing the financial burden of medication expenses. This article has compiled studies like systematic reviews, meta-analyses, and randomized controlled trials and reviewed the role of non-pharmacological management of HTN, including lifestyle modifications like exercise, weight loss, dietary interventions like dietary approaches to stop hypertension (DASH) diet, low sodium diet, limiting alcohol consumption, smoking cessation and stress management to help control blood pressure. However, non-pharmacological interventions should be initiated in an early phase, and for proper management of HTN, we may need to include both pharmacological and non-pharmacological interventions. This article explored and integrated various studies to highlight the role of non-pharmacological interventions to manage HTN. We also examined the need for more research studies for strategies to alleviate morbidity and mortality associated with HTN.

## Introduction and background

By 2025, hypertension (HTN) is estimated to affect one-third of the world's population [1]. According to the American College of Cardiology and the American Heart Association, HTN is an elevated blood pressure (BP) reading of 130/80 mm Hg or higher. It was in 1896 that HTN was first recognized as a clinical entity with the invention of the cuff-based mercury sphygmomanometer by Italian physician Scipione Riva-Rocci [2].

According to the American College of Cardiology and the American Heart Association, BP is divided into four general categories (Table 1).

**Table 1:** Classification of blood pressure. BP: blood pressure, HTN: hypertension.

Category	Blood pressure
Normal BP	120/80 mm Hg or lower
Elevated BP	Systolic from 120 to 129 mm Hg and diastolic below, not above, 80 mm Hg
Stage 1 HTN	Systolic from 130 to 139 mm Hg or diastolic between 80- and 89-mm Hg
Stage 2 HTN	Systolic 140 mm Hg or higher or diastolic 90 mm Hg or higher

HTN affects people with advancing age. It is more common in ethnically African and South Asian communities [3]. Major risk factors include family history, advancing age, high-sodium diet, obesity, alcohol consumption, and physical inactivity. HTN is rarely symptomatic when BP is very high. Some symptoms include blurry vision, shortness of breath, and headache. In addition to advanced age, obesity, and other risk factors, positive genes are associated with nearly 50% of primary hypertensives [4].

According to the American College of Cardiology, a BP higher than 180/120 mm Hg is considered a hypertensive emergency or crisis. Patients with these blood pressures need emergency medical help. Untreated high BP may increase the risk of myocardial infarction, stroke, and other serious complications. Monitoring BP every two years, starting at age 18, is important to diagnose and treat hypertension timely to prevent complications. HTN is diagnosed by performing repeated careful measurements of blood pressure. Blood pressure is categorized as follows: Normal blood pressure, defined as systolic blood pressure (SBP) less than 120, and diastolic blood pressure (DBP) less than 80. An elevated BP is an SBP of 120 to 129 and a DBP of less than 80. HTN is defined as a systolic pressure more than or equal to 130 or a diastolic pressure more than or equal to 80.

One of the quintessential steps to manage HTN is lifestyle modifications like exercise, weight loss, dietary interventions, a low-sodium diet, limiting alcohol consumption, smoking cessation, and stress management to help control BP. The treatment of HTN is based on specific characteristics like stage of disease, compliance, and comorbid conditions. For proper management of HTN, we may need to include pharmacological and non-pharmacological interventions [5]. Despite advancements in medicine and treatment options, the global burden of HTN has been increasing due to the advancing age of the population and an increasing prevalence of obesity. HTN is an insidious disease that, if not treated promptly, predisposes us to cardiovascular complications and various other complications [6]. Increased physical activity, limiting salt intake, minimizing alcohol consumption, smoking cessation, and stress management together support the management of patients with HTN and as preventive measures in the general population [7]. However, it is important to note that lifestyle modification is a process that requires patients to adhere continuously [5].

This article aims to emphasize the non-pharmacological management of HTN as it is an attractive approach to dealing with HTN in developed and developing countries due to the economic limitations and having additional benefits with few or almost no side effects [6]. Another goal of the article is to underline the importance of non-pharmacological interventions to help reduce the daily dose of antihypertensive medication and to delay the progression from prehypertension to the HTN stage [5]. This article also intends to vocalize the effect of dietary approaches, such as dietary approaches to stop hypertension (DASH)-like diet, on reducing both systolic and diastolic BP in adults [8]. 

## Review

High BP can lead to complications like coronary artery disease, congestive heart failure, end-stage renal disease, and stroke [9]. A study by Ghadeih et al. performed in 2014 revealed that with each 20 mm increase of systolic BP above normal, the risk of cardiovascular events doubles [9]. In the modern world, HTN is a common disorder, and more than 86 million adults are hypertensive in the United States [10].

Considering aspects like cost, effectiveness, and the potential for side effects of antihypertensive medications, lifestyle interventions (Table 2), including weight loss, exercise, diet, lowering alcohol consumption and smoking, and managing stress, have gained emphasis on treating and preventing HTN [9]. 

**Table 2:** Impact of non-pharmacological interventions on systolic blood pressure of hypertensive versus normotensive individuals. SBP: systolic blood pressure; DBP: diastolic blood pressure; HTN: hypertension; DASH: dietary approaches to stop hypertension.

Non-pharmacological intervention	Impact on SBP in individuals with HTN	Impact on SBP in normotensive individuals
Weight loss [11]	−5 mm Hg	−2/3 mm Hg
DASH diet [11]	−11 mm Hg	−3 mm Hg
Low-sodium diet [11]	−5/6 mm Hg	−2/3 mm Hg
Increased dietary potassium [11]	−4/5 mm Hg	−2 mm Hg
Exercise [11]	−4 to 8 mm Hg	−2 to 4 mm Hg
Lowering alcohol consumption [11]	−4 mm Hg	−3 mm Hg

Weight loss

Weight loss is one of the most important non-pharmacological interventions to lower BP. Numerous interrelated pathophysiologic mechanisms stimulate higher BP in obesity [12]. In overweight/obese individuals, accelerated vascular aging can lead to HTN due to inflammation, oxidative stress, and insulin resistance [13]. Obese individuals also experience increased activity of the sympathetic nervous system and the renin-angiotensin-aldosterone system [14]. The combined effect results in increased sodium resorption by the kidney, impaired vasodilation, volume expansion, and decreased natriuresis, thus leading to elevated BP [15]. Ozemek et al. trials have shown that weight loss helped lower systolic BP in hypertensive individuals by 5 mm Hg and in normotensive individuals by 2 to 3 mm Hg. According to the Centers for Disease Control (CDC), a BMI of 25 to 29.9 is considered overweight, and a BMI of 30 or higher is considered obese. In overweight or obese individuals, achieving their ideal body weight is best, but it is good to aim for at least a 1 kg reduction in body weight. The study by Ozemek et al. also revealed that for every 1 kg reduction in body weight, we can expect about a 1 mm Hg reduction in blood pressure. 

Intermittent fasting (IF) is an effective way to lose weight and thus helps lower blood pressure [10]. The mechanism by which IF lowers BP may be due to a brain-derived neurotrophic factor (BDNF)-induced increase in parasympathetic activity [16]. Increased excretion of norepinephrine and increased sensitivity of insulin and natriuretic peptides also play a role [16]. The activation of glutamatergic receptors produces BDNF [10]. IF also stimulates the release of BDNF [10]. BDNF, in turn, stimulates the cholinergic neurons to release acetylcholine, which via the vagus nerve, controls the cardiac function to the sinoatrial (SA) node, causing a reduction of heart rate [17]. Also, blood vessels are expanded by the neurotransmitter, leading to a reduction in BP [18]. The pathogenesis of blood pressure lowering by activation of the parasympathetic nervous system involves the role of the cerebrospinal stem in the activation of cholinergic neurons [19,20]. However, cardiovascular health benefits have only been observed to last as long as the IF diet lasted and pressures returned to initial values after the completion of the IF diet [21]. Toledo et al. performed a study in Germany in which 1422 participants on the IF diet were followed up for one year [16]. These participants had a fasting period of four to 21 days, which involved 200-250 kcal daily meals [16]. In participants who fasted for a longer time, a reduction of SBP and DBP was observed [16].

Assorted studies have shown that IF lowers BP. Harvie et al., in their study involving 107 overweight or obese premenopausal women, showed that IF for six months helped lower SBP (p = 0.99) and DBP (p = 0.84) [22]. Varady et al., in their study performed for 12 weeks with IF involving 15 overweight individuals (five males, 10 females with a BMI of 20-29.9 kg/m^2^ showed that IF helped lower BP with a p-value of 0.51 [23]. A study by Bhutani et al. involving 83 obese individuals (three males and 80 females) with a BMI of 30-39.9 kg/m^2^ revealed that 12 weeks of IF helped lower SBP (p = 0.254) and DBP (p = 0.570) [24]. In a study by Eshghinia et al., 15 overweight or obese women with a BMI ≥25 kg/m^2^ who followed IF for eight weeks showed a lowering of SBP (p<0.001) and DBP (p<0.05) [25]. Teng et al., in their 12 weeks IF study with 28 Malay men with a BMI of 23-29.9 kg/m^2^, showed a lowering in SBP (p<0.05) and DBP (p<0.05) [26]. Erdem et al., in their study involving 60 participants from the Cappadocia cohort with pre-HTN and HTN with SBP of 120-139 and more than or equal to 140 mm Hg, DBP of 80-80 and more than or equal to 90 mm Hg, revealed that IF helps lower SBP (p<0.001) and DBP (p<0.039) [27]. 

Practicality of Implementation

Although weight loss plays a significant role in lowering BP, it may be challenging to stay constantly motivated for long-term results [28]. Developing strategies to identify individuals who are unable to maintain lifestyle changes may be crucial to help them stay motivated to achieve weight management goals [28]. Furthermore, in recent times, an effective tool to promote the maintenance of healthy lifestyle changes like weight loss can be the use of mobile technology and personal digital devices, especially when individuals no longer have the availability of support and accountability through active interventions [29]. In hypertensive individuals with normal weight, other interventions like the DASH diet help lower BP [28].

Diet

After originating in the 1990s, the dietary approaches to stop hypertension gained popularity in lowering blood pressure [30]. The DASH dietary pattern involves the consumption of a diet rich in fruits, vegetables, whole grains, low-fat dietary products, and low saturated and total fat [11]. The DASH diet also focuses attention on the reduction of sodium in the diet and recommends avoiding the consumption of processed foods [30]. The mechanisms involved in salt-dependent increased blood pressure include volume expansion, disorders in sodium balance, modified renal functions, impaired renin-angiotensin-aldosterone system, central stimulation of the sympathetic nervous system, and inflammatory processes [31].

The DASH diet has been funded by the National Institute of Health (NIH) in various research projects to know whether specific dietary interventions helped treat HTN [30]. The subjects included in the study have been told to follow only dietary interventions and no other lifestyle modifications to avoid any confounding [30]. It was found in both hypertensive and normotensive individuals that dietary intervention alone helped reduce SBP by 6 to 11 mm Hg [30]. Since these results, the DASH diet, along with lifestyle modifications, has been advised as the first line of pharmacologic therapy in some instances. Several other clinical trials have shown that the DASH diet helps lower BP, cholesterol, and saturated fats as well as there is evidence that it lowers the risk of adverse cardiac events, type 2 diabetes, stroke, and obesity [30]. Therefore, it is crucial for clinicians, nurses, and pharmacists to educate patients about the benefits of the DASH diet [30]. 

DASH recommends the consumption of: Healthy carbohydrates like green leafy vegetables (broccoli, spinach, kale, mustards, collards), whole grains (cracked wheat, oats, millets), low-glycemic-index fruits, legumes, and beans [30]; Healthy fats like olive oil, avocados, nuts, hemp seeds, flax seeds, and fish rich in omega-3 fatty acids [30]; Healthy proteins like plant proteins (legumes, soy proteins, nuts, seeds) and animal proteins, including lean meat, low-fat dairy, eggs, and fish [30]; Foods rich in potassium (bananas, oranges, spinach), calcium (dairy, green leafy vegetables), and magnesium (whole grains, leafy vegetables, nuts, seeds) [30].

According to a study by Ozemek et al., healthy diets like DASH dietary patterns have been shown to decrease SBP by 11 mm Hg in hypertensive individuals and by 3 mm Hg in normotensive individuals. Ideally, the goal is to reduce sodium to <1500 mg/dl, but it is good to aim for at least a 1000 mg/day reduction, which has been shown to reduce SBP approximately by 5/6 mm Hg in hypertensive individuals and by 2/3 mm Hg in normotensive individuals [11]. Furthermore, Ozemek et al. revealed in their study that increasing dietary potassium intake with a goal of 3500-5000 mg/day has been shown to reduce SBP by −4/5 mm Hg in hypertensive individuals and by −2 mm Hg in normotensive individuals [11].

Hinderliter et al. performed the Exercise and Nutrition Interventions for Cardiovascular Health (ENCORE) study, a trial that involved observing the persistence of lifestyle modifications in 144 overweight participants with higher-than-normal BP for 16 weeks, randomly assigned to the DASH diet alone (DASH-A), DASH diet plus weight management (DASH-WM), or usual care [28]. Individuals were followed for eight months after the end of the assigned intervention. Compared to baseline, at one year, SBP showed a reduction of 11.7 mm Hg in the DASH-WM, 9.5 mm Hg in the DASH-A, and 3.9 mm Hg in usual care [28]. Even after 48 months of discontinuing individuals' contact with the clinical center, the intervention had a long-term effect on BP, resulting in 23% of the combined intervention group staying off medication vs. 7% of the usual care group [28]. The DASH diet has similarities to some other dietary designs that have a positive impact on the cardiovascular system [30].

The Mediterranean diet (Med Diet) has also helped lower BP. As per the Maine-Syracuse Longitudinal Study conducted in the United States in 2020 by a group of researchers who followed 851 US older adults, for every one unit increase in the Med Diet score in participants, it was found that there was a corresponding reduction of 0.69 units in SBP, a reduction of 0.33 in DBP, and a reduction of 0.45 on mean arterial pressure (MAP) [32]. Although this seems to be small, this change can have a noteworthy effect at the level of the population; that is, a decrease of 2 mm Hg in SBP can lead to a decrease of 10% when it comes to the population [32]. According to the observational studies conducted in Mediterranean countries, higher adherence to a Med Diet is associated with a decreased risk of cardiovascular disease, overall mortality as well as neoplastic disease [33,34]. The Med Diet consists of higher consumption of extra virgin olive oil, vegetables, fruits, whole grains, nuts, cereals, as well as seeds; moderate consumption of fish, poultry, red wine, and dairy; and lower consumption of processed foods and red meat [35].

Practicality of Implementation

Patients can find it increasingly difficult to maintain long-term dietary changes like low caloric intake, low salt intake, and limiting processed foods, which in turn may lead to a shift towards preintervention practices in some individuals, for example, increased calorie intake [28]. This implies the need for interventions to focus on ways to help patients maintain healthy dietary patterns [28].

Exercise

Increased physical activity has been advocated as the first-line intervention for preventing and treating patients with prehypertension and as a treatment strategy for patients with stage 1 or stage 2 HTN, according to the Duthe American College of Sports Medicine, the United States Joint Nations Committee on Prevention, Detection, Evaluation, and Treatment of High BP, the World Health Organization and International Society of Hypertension, and The National Heart Foundation [36]. Exercise can consequently help prevent prehypertension from progressing and can help reduce or stop medications prescribed for the treatment of stage 1 HTN [37]. 

The pathogenesis of HTN involves oxidative stress. Another mechanism involved is the decreased bioavailability of nitric oxide (NO) [38]. Physical exercise could be a potential lifestyle intervention to treat HTN due to its beneficial effects on endothelial function and oxidative stress [38]. Exercise exerts an anti-inflammatory action via the hypothalamic-pituitary-adrenal axis and via the sympathetic nervous system, thus affecting BP directly [9]. The physiologic effects of exercise are further divided into acute, post-exercise, and chronic [9]. Aerobic exercises like speed walking, jogging, running, cycling, dancing, and swimming have been shown to decrease resting BP and BP reactivity to stressors [9]. A study by Ozemek et al. revealed the following about how diverse types of exercises affect BP: (1) Aerobic exercise of 90 to 150 minutes per week with 65%-75% heart rate reserve has been shown to impact SBP by −5/8 mm Hg in hypertensive individuals and by −2/4 mm Hg in normotensive individuals. (2) Dynamic resistance exercise of 90 to 150 minutes per week with 50%-80% one rep maximum, six exercises, three sets/exercise, and ten repetitions/set has been shown to decrease SBP by 4 mm Hg in hypertensive individuals and 2 mm Hg in normotensive individuals. (3) Isometric resistance exercise of 4 × 2 min (hand grip), 1 min rest between exercises, 30%-40% maximum voluntary contraction, and three sessions per week for 8-10 weeks have been shown to lower SBP by 5 mm Hg in hypertensive individuals and 4 mm Hg in normotensive individuals [11].

The following four randomized controlled trials have been conducted to show that exercise helped lower both systolic and diastolic BP in participants: In the meta-analyses of randomized controlled trials conducted by Fagard et al., 72 trials have been conducted with an average of 40 participants per trial, which involved a 16-week study of 40 minutes of exercise sessions three times/week with an average intensity of 65% of heart rate [39]. This study showed a decrease in SBP of 6.9 mm Hg and a lowering of DBP of 4.9 mm Hg [39]. In 27 randomized controlled trials by Lee et al. with 1842 participants, the exercise regimen involved walking for 26.5 min/day for 4.4 days/week for a mean of 19 weeks [40]. This study shows that there is a larger effect with more intense and frequent exercise regimens for a longer duration [40]. There was a mean decrease in SBP of 5.2 to 11 mm Hg and in DBP of 3.8 to 7.7 mm Hg [40]. In the meta-analysis of randomized control trials performed by Cornelissen et al., 15 trials with 633 participants involving the exercise of 30-60 min, two to five times/week, at 50% to 75% HR reserve for six to 52 weeks showed a daytime decrease in SBP of 3.2 mm Hg and in DBP of 2.7 mm Hg [41]. However, no blood pressure reduction was seen at night [42]. The meta-analysis of randomized controlled trials performed by Cornelissen and Smart included 105 trials with 3957 participants [42]. This study concluded that moderate aerobic exercise involving walking and jogging for 30 to 60 min/session three to five times/week for four to 52 weeks showed a reduction in SBP of 3.5 mm Hg and in DBP of 2.5 mm Hg [42].

*Practicality of Implementation* 

Patients may be initially motivated to engage in exercise programs but can find it challenging in long-term maintenance, which may lead to weight regain [43]. In order to achieve effective weight loss, apart from exercise, individuals should follow caloric restrictions [44]. Furthermore, patients may feel discouraged if they do not achieve desired weight loss despite exercising; therefore, clinicians should explain and strongly encourage patients regarding long-term adherence to exercise training programs despite the achieved amount of weight loss as regardless of achieving weight loss, exercise helps achieve cardiovascular benefits [44].

Alcohol

The health effects of alcohol intake are variable and are based on the amount of intake (low, moderate, or heavy) and intake pattern (acute, chronic, or binge) [45-47]. In the US, the amount of pure alcohol in one standard drink is 14 g, which can be found in 12 oz of regular beer (about 5% alcohol), 5 oz of wine (about 12% alcohol), and 1.5 oz of distilled spirits (about 40% alcohol) [48]. Moderate alcohol intake recommendations according to the Dietary Guidelines for Americans are the consumption of two standard drinks per day for men and one standard drink per day for women [49]. Moderate alcoholic intake has been shown to reduce the risk of chronic disease [49]. 

According to the National Institute on Alcohol Abuse and Alcoholism (NIAAA), low-risk drinking is considered four drinks per day or less than fourteen drinks per week for men and less than or equal to three drinks per day and less than seven drinks per day for women [49]. Binge drinking is the consumption of five drinks by men and four drinks by women in a period of two hours [47,50]. According to studies performed by O’Keefe et al. and Peng et al., it was shown that excessive consumption of alcohol has contributed to 16% of HTN cases worldwide [45,51]. The pathophysiology of HTN in chronic alcohol consumption is by vagal inhibition and sympathetic stimulation [52]. 

According to a study performed by Fuchs et al. in 2021, it has been found that, especially in women, the risk has been found to start at moderate alcohol consumption, and alcohol withdrawal has been found to promote the reduction in BP in short-term trials [52]. Suliga et al., in their cross-sectional study of 12,285 individuals in the age group 37 to 66, described that daily alcohol consumption ranging from 0.1 g to 15 g was inversely related to the development of HTN in women, with an odds ratio of 0.67, 95% confidence interval of 0.59 to 0.75, p-value <0.001 [53]. However, in men, this relation was not observed [53]. 

Briasoulis et al., in their meta-analysis of 16 prospective long-term studies, revealed that alcohol consumption of more than 20 g per day increases the risk of HTN significantly in women, whereas consumption of 31 to 40 g in men significantly increases the risk of HTN [54]. Nevertheless, a higher risk of HTN was observed in all individuals who consumed more than 20 g of alcohol per day, irrespective of their gender [54]. Ozemek et al. revealed in their study that, in persons who consume alcohol, a reduction to two standard drinks or less daily in men and one drink or less daily in women has been shown to reduce SBP by 4 mm Hg in hypertensive individuals and by 3 mm Hg in normotensive individuals [11].

It should be taken into consideration that the effects of alcohol are not similar in all individuals [49]. Also, individuals with multiple co-existing risk factors like a sedentary lifestyle, lack of exercise, overweight or obesity, smoking, along with excessive alcohol consumption have higher cardiovascular risks [55]. 

Smoking

**Figure 1:**
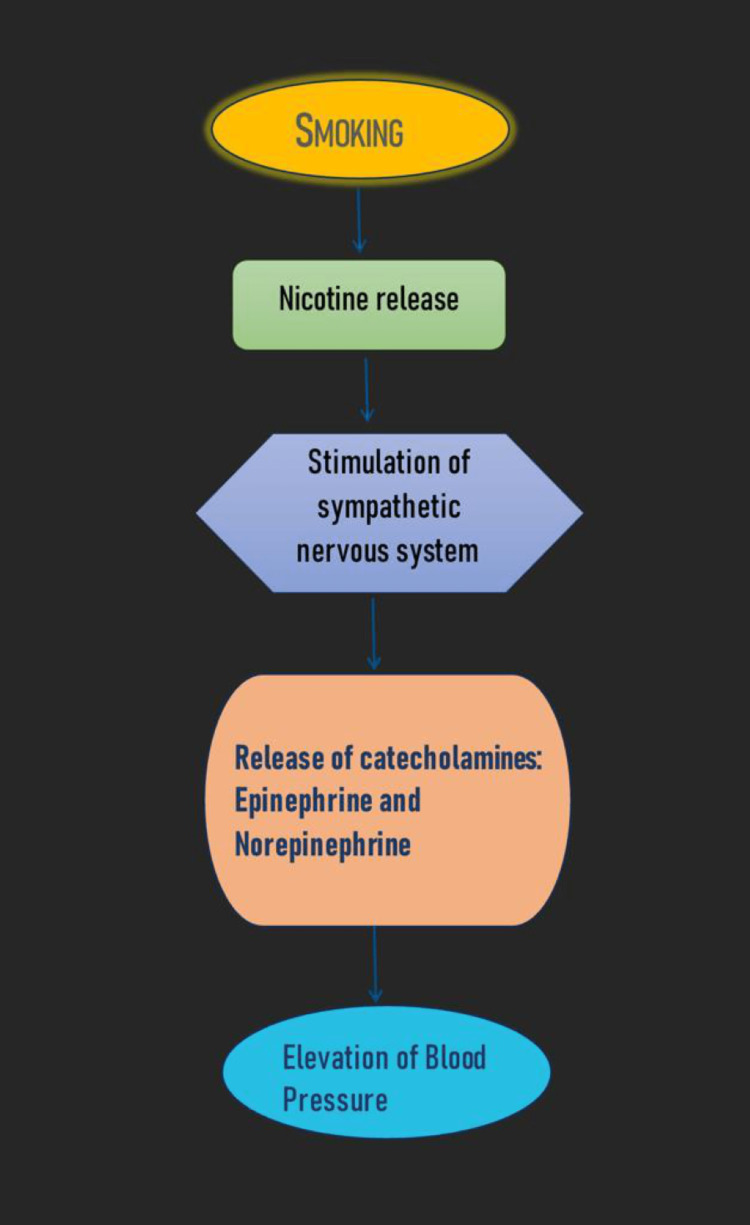
Mechanism of blood pressure elevation by smoking. Image is created by own author's creation.

Smoking is an important risk factor for HTN. The following is the mechanism involved in the elevation of BP by smoking (Figure 1).

Physicians play a key role in encouraging and helping patients achieve smoking cessation [56]. HTN has also been associated with second-hand smoke. According to research done by Bernabe-Ortiz et al. on 897 individuals in Peru in 2021 to assess the association of second-hand smoke with HTN and cardiovascular risk, 15% of adults reported second-hand smoke overall, and this emphasizes the necessity to keep places smoke-free to reduce the risk of cardiovascular disease [57].

Psychological stress

Psychological stress is another contributor to HTN [58]. Stress can induce neuroendocrine responses after being perceived by the brain, which can lead to inflammation and endothelial dysfunction, which in turn modulate the elevation of BP with stress [58]. A range of factors like globalization, socioeconomic changes, stress at the workplace, and cultural changes have led to an increased number of people experiencing chronic psychological stress in recent times [59]. It is important to take into consideration the role of psychological stress in the pathogenesis of elevated BP in order to arrive at an individual-focused approach [58]. Meditation, yoga, and other stress management techniques like acupuncture, tai chi, and mindfulness-based stress reduction programs have been demonstrated to be effective in decreasing BP, but this effect is variable in different age groups and differences in BPs of different individuals; however, these practices are considered safe alternatives in some cases [59,60].

Limitations

In this study, we focused on HTN as a single factor and outlined the management; however, we have not taken into consideration other comorbidities like diabetes and hyperlipidemia that may co-exist with HTN. Although various impactful studies were integrated, this study could not examine and evaluate all the available data. This study does not address the challenges with adherence to lifestyle modifications like exercise and dietary interventions, as well as the challenges with compliance with limiting alcohol and cessation of smoking. Although non-pharmacological management alone can be effective in patients with prehypertension or stage 1 HTN, however, for stage 2 HTN, pharmacological management is still considered the first line. Furthermore, this study did not outline the combined effects of medications and lifestyle modifications.

## Conclusions

It is evident that uncontrolled HTN can lead to serious complications like myocardial infarction, stroke, end-stage renal disease, and other manifestations of end-organ damage. In summary, the clinical implication of this review article is to establish a strong emphasis on the management of essential HTN with non-pharmacological interventions involving multiple elements of lifestyle modification, focusing on more permanent measures to help improve the overall quality of life and to decrease physician dependence and hospitalizations for serious complications. Weight loss is the most beneficial non-pharmacological intervention to manage HTN, followed by the DASH diet, exercise, alcohol, smoking, and management of stress. These should be initiated in the early phase and used along with pharmacological intervention only to be highly effective. Clinicians play a crucial role in explaining the risks of untreated HTN to patients and strongly encouraging them regarding long-term adherence to lifestyle interventions like weight loss, exercise, lowering alcohol consumption, and smoking cessation. The use of mobile technology and personal digital devices can be effective to help track and maintain healthy lifestyle changes like weight loss and exercise, especially when support and accountability through active intervention are not available. Even though various studies have been performed, more in-depth research studies need to be conducted to master the approach to minimize morbidity and mortality associated with HTN.

## References

[1] Oliveros E, Patel H, Kyung S, Fugar S, Goldberg A, Madan N, Williams KA (2020). Hypertension in older adults: assessment, management, and challenges. Clin Cardiol.

[2] (2017). Harold on history. Historical perspectives on hypertension. https://www.acc.org/latest-in-cardiology/articles/2017/11/14/14/42/harold-on-history-historical-perspectives-on-hypertension.

[3] Dwivedi G, Beevers DG (2009). Hypertension in ethnic groups: epidemiological and clinical perspectives. Expert Rev Cardiovasc Ther.

[4] Manosroi W, Williams GH (2019). Genetics of human primary hypertension: focus on hormonal mechanisms. Endocr Rev.

[5] Mahmood S, Shah KU, Khan TM, Nawaz S, Rashid H, Baqar SW, Kamran S (2019). Non-pharmacological management of hypertension: in the light of current research. Ir J Med Sci.

[6] Verma N, Rastogi S, Chia YC (2021). Non-pharmacological management of hypertension. J Clin Hypertens (Greenwich).

[7] Maniero C, Lopuszko A, Papalois KB, Gupta A, Kapil V, Khanji MY (2023). Non-pharmacological factors for hypertension management: a systematic review of international guidelines. Eur J Prev Cardiol.

[8] Saneei P, Salehi-Abargouei A, Esmaillzadeh A, Azadbakht L (2014). Influence of Dietary Approaches to Stop Hypertension (DASH) diet on blood pressure: a systematic review and meta-analysis on randomized controlled trials. Nutr Metab Cardiovasc Dis.

[9] Ghadieh AS, Saab B (2015). Evidence for exercise training in the management of hypertension in adults. Can Fam Physician.

[10] Malinowski B, Zalewska K, Węsierska A (2019). Intermittent fasting in cardiovascular disorders-an overview. Nutrients.

[11] Ozemek C, Tiwari S, Sabbahi A, Carbone S, Lavie CJ (2020). Impact of therapeutic lifestyle changes in resistant hypertension. Prog Cardiovasc Dis.

[12] Cohen JB, Gadde KM (2019). Weight loss medications in the treatment of obesity and hypertension. Curr Hypertens Rep.

[13] Sironi AM, Gastaldelli A, Mari A (2004). Visceral fat in hypertension: influence on insulin resistance and beta-cell function. Hypertension.

[14] Buglioni A, Cannone V, Cataliotti A (2015). Circulating aldosterone and natriuretic peptides in the general community: relationship to cardiorenal and metabolic disease. Hypertension.

[15] Lohmeier TE, Iliescu R (2013). The sympathetic nervous system in obesity hypertension. Curr Hypertens Rep.

[16] Wilhelmi de Toledo F, Grundler F, Bergouignan A, Drinda S, Michalsen A (2019). Safety, health improvement and well-being during a 4 to 21-day fasting period in an observational study including 1422 subjects. PLoS One.

[17] Yang B, Slonimsky JD, Birren SJ (2002). A rapid switch in sympathetic neurotransmitter release properties mediated by the p75 receptor. Nat Neurosci.

[18] Wang J, Irnaten M, Neff RA (2001). Synaptic and neurotransmitter activation of cardiac vagal neurons in the nucleus ambiguus. Ann N Y Acad Sci.

[19] Mattson MP, Longo VD, Harvie M (2017). Impact of intermittent fasting on health and disease processes. Ageing Res Rev.

[20] Wan R, Weigand LA, Bateman R, Griffioen K, Mendelowitz D, Mattson MP (2014). Evidence that BDNF regulates heart rate by a mechanism involving increased brainstem parasympathetic neuron excitability. J Neurochem.

[21] Mager DE, Wan R, Brown M, Cheng A, Wareski P, Abernethy DR, Mattson MP (2006). Caloric restriction and intermittent fasting alter spectral measures of heart rate and blood pressure variability in rats. FASEB J.

[22] Harvie MN, Pegington M, Mattson MP (2011). The effects of intermittent or continuous energy restriction on weight loss and metabolic disease risk markers: a randomized trial in young overweight women. Int J Obes.

[23] Varady KA, Bhutani S, Klempel MC (2013). Alternate day fasting for weight loss in normal weight and overweight subjects: a randomized controlled trial. Nutr J.

[24] Bhutani S, Klempel MC, Kroeger CM, Trepanowski JF, Varady KA (2013). Alternate day fasting and endurance exercise combine to reduce body weight and favorably alter plasma lipids in obese humans. Obesity (Silver Spring).

[25] Eshghinia S, Mohammadzadeh F (2013). The effects of modified alternate-day fasting diet on weight loss and CAD risk factors in overweight and obese women. J Diabetes Metab Disord.

[26] Teng NI, Shahar S, Rajab NF, Manaf ZA, Johari MH, Ngah WZ (2013). Improvement of metabolic parameters in healthy older adult men following a fasting calorie restriction intervention. Aging Male.

[27] Erdem Y, Özkan G, Ulusoy Ş (2018). The effect of intermittent fasting on blood pressure variability in patients with newly diagnosed hypertension or prehypertension. J Am Soc Hypertens.

[28] Hinderliter AL, Sherwood A, Craighead LW, Lin PH, Watkins L, Babyak MA, Blumenthal JA (2014). The long-term effects of lifestyle change on blood pressure: one-year follow-up of the ENCORE study. Am J Hypertens.

[29] Spring B, Duncan JM, Janke EA (2013). Integrating technology into standard weight loss treatment: a randomized controlled trial. JAMA Intern Med.

[30] Challa HJ, Ameer MA, Uppaluri KR (2023). DASH diet to stop hypertension. StatPearls (Internet).

[31] Rust P, Ekmekcioglu C (2017). Impact of salt intake on the pathogenesis and treatment of hypertension. Adv Exp Med Biol.

[32] Ahmed FS, Wade AT, Guenther BA, Murphy KJ, Elias MF (2020). Adherence to a Mediterranean diet associated with lower blood pressure in a US sample: findings from the Maine-Syracuse Longitudinal Study. J Clin Hypertens (Greenwich).

[33] Sofi F, Macchi C, Abbate R, Gensini GF, Casini A (2014). Mediterranean diet and health status: an updated meta-analysis and a proposal for a literature-based adherence score. Public Health Nutr.

[34] Trichopoulou A, Costacou T, Bamia C, Trichopoulos D (2003). Adherence to a Mediterranean diet and survival in a Greek population. N Engl J Med.

[35] Davis C, Bryan J, Hodgson J, Murphy K (2015). Definition of the Mediterranean diet; a literature review. Nutrients.

[36] Baster T, Baster-Brooks C (2005). Exercise and hypertension. Aust Fam Physician.

[37] Sainani GS (2003). Non-drug therapy in prevention and control of hypertension. J Assoc Physicians India.

[38] Korsager Larsen M, Matchkov VV (2016). Hypertension and physical exercise: the role of oxidative stress. Medicina (Kaunas).

[39] Fagard RH, Cornelissen VA (2007). Effect of exercise on blood pressure control in hypertensive patients. Eur J Cardiovasc Prev Rehabil.

[40] Lee LL, Watson MC, Mulvaney CA, Tsai CC, Lo SF (2010). The effect of walking intervention on blood pressure control: a systematic review. Int J Nurs Stud.

[41] Cornelissen VA, Buys R, Smart NA (2013). Endurance exercise beneficially affects ambulatory blood pressure: a systematic review and meta-analysis. J Hypertens.

[42] Cornelissen VA, Smart NA (2013). Exercise training for blood pressure: a systematic review and meta-analysis. J Am Heart Assoc.

[43] Lisón JF, Palomar G, Mensorio MS (2020). Impact of a web-based exercise and nutritional education intervention in patients who are obese with hypertension: randomized wait-list controlled trial. J Med Internet Res.

[44] Swift DL, Johannsen NM, Lavie CJ, Earnest CP, Church TS (2014). The role of exercise and physical activity in weight loss and maintenance. Prog Cardiovasc Dis.

[45] O'Keefe EL, DiNicolantonio JJ, O'Keefe JH, Lavie CJ (2018). Alcohol and CV health: Jekyll and Hyde J-Curves. Prog Cardiovasc Dis.

[46] Piano MR (2017). Alcohol's effects on the cardiovascular system. Alcohol Res.

[47] Alcohol Research: Current Reviews Editorial Staff (2018). Drinking patterns and their definitions. Alcohol Res.

[48] Arnett DK, Blumenthal RS, Albert MA (2019). 2019 ACC/AHA guideline on the primary prevention of cardiovascular disease: a report of the American College of Cardiology/American Heart Association Task Force on Clinical Practice Guidelines. Circulation.

[49] Minzer S, Losno RA, Casas R (2020). The effect of alcohol on cardiovascular risk factors: is there new information?. Nutrients.

[50] Chiva-Blanch G, Arranz S, Lamuela-Raventos RM, Estruch R (2013). Effects of wine, alcohol and polyphenols on cardiovascular disease risk factors: evidences from human studies. Alcohol Alcohol.

[51] Peng M, Wu S, Jiang X, Jin C, Zhang W (2013). Long-term alcohol consumption is an independent risk factor of hypertension development in northern China: evidence from Kailuan study. J Hypertens.

[52] Fuchs FD, Fuchs SC (2021). The effect of alcohol on blood pressure and hypertension. Curr Hypertens Rep.

[53] Suliga E, Kozieł D, Ciesla E, Rebak D, Głuszek-Osuch M, Naszydłowska E, Głuszek S (2019). The consumption of alcoholic beverages and the prevalence of cardiovascular diseases in men and women: a cross-sectional study. Nutrients.

[54] Briasoulis A, Agarwal V, Messerli FH (2012). Alcohol consumption and the risk of hypertension in men and women: a systematic review and meta-analysis. J Clin Hypertens (Greenwich).

[55] Loef M, Walach H (2012). The combined effects of healthy lifestyle behaviors on all cause mortality: a systematic review and meta-analysis. Prev Med.

[56] Samadian F, Dalili N, Jamalian A (2016). Lifestyle modifications to prevent and control hypertension. Iran J Kidney Dis.

[57] Bernabe-Ortiz A, Carrillo-Larco RM (2021). Second-hand smoking, hypertension and cardiovascular risk: findings from Peru. BMC Cardiovasc Disord.

[58] Munakata M (2018). Clinical significance of stress-related increase in blood pressure: current evidence in office and out-of-office settings. Hypertens Res.

[59] Liu MY, Li N, Li WA, Khan H (2017). Association between psychosocial stress and hypertension: a systematic review and meta-analysis. Neurol Res.

[60] Park SH, Han KS (2017). Blood pressure response to meditation and yoga: a systematic review and meta-analysis. J Altern Complement Med.

